# Expression of the Tpl2/Cot oncogene in human T-cell neoplasias

**DOI:** 10.1186/1476-4598-3-34

**Published:** 2004-12-03

**Authors:** Anna V Christoforidou, Helen A Papadaki, Andrew N Margioris, George D Eliopoulos, Christos Tsatsanis

**Affiliations:** 1 Department of Clinical Chemistry-Biochemistry, School of Medicine, University of Crete and University Hospital of Heraklion, 71110 Heraklion, Crete, Greece; 2 Department of Hematology, School of Medicine, University of Crete and University Hospital of Heraclion, 71110 Heraclion, Crete, Greece

## Abstract

**Background:**

Tpl2/Cot oncogene has been identified in murine T-cell lymphomas as a target of MoMuLV insertion. Animal and tissue culture studies have shown that Tpl2/Cot is involved in interleukin-2 (IL-2) and tumor necrosis factor-α (TNF-α) production by T-cells contributing to T-cell proliferation. In the present report we examined a series of 12 adult patients with various T-cell malignancies, all with predominant leukemic expression in the periphery, for the expression of Tpl2/Cot oncogene in order to determine a possible involvement of Tpl2/Cot in the pathogenesis of these neoplasms.

**Results:**

Our results showed that Tpl2/Cot was overexpressed in all four patients with Large Granular Lymphocyte proliferative disorders (LGL-PDs) but in none of the remaining eight patients with other T-cell neoplasias. Interestingly, three of the LGL-PD patients displayed neutropenia, one in association with sarcoidosis. Serum TNF-α levels were increased in all Tpl2/Cot overexpressing patients while serum IL-2 was undetectable in all subjects studied. Genomic DNA analysis revealed no DNA amplification at the Tpl2/Cot locus in any of the samples analyzed.

**Conclusions:**

We conclude that Tpl2/Cot, a gene extensively studied in animal and tissue culture T-cell models may be also involved in the development of human LGL-PD and may have a role in the pathogenesis of immune manifestations associated with these diseases. This is the first report implicating Tpl2/Cot in human T-cell neoplasias and provides a novel molecular event in the development of LGL-PDs.

## Background

Cells may transform to a malignant phenotype following accumulation of distinct genetic events that result in altered protein expression pattern, thus facilitating uncontrolled proliferation. Such genetic events target specific oncogenes that act in concert to provide the malignant phenotype.

Tpl2/Cot oncogene was initially cloned as a MoMuLV proviral integration locus in murine T-cell lymphoma cells, resulting in its carboxy-terminal truncation[1,2]. Expression of the truncated form of Tpl2 as a transgene in T-cells under the control of the *lck *promoter in mice results in rapid development of T-cell lymphomas [3]. Expression of Tpl2 is associated with T-cell activation. Overexpression of the wild type Tpl2 in the Jurkat T-cell leukemia cell line results in NFkB and NFAT activation and subsequent IL-2 and TNF-α expression [4-7]. In the CTLL2 IL-2 dependent cell line Tpl2 promotes cell proliferation by activating E2F-dependent transcription [8]. Tpl2/Cot is, therefore, tightly associated with T-cell neoplasms and T-cell activation and proliferation.

Studies in human tumor specimens have shown that Tpl2/Cot is overexpressed in early stage breast cancer [9], in EBV-related Hodgkin lymphomas and nasopharyngeal carcinomas [10] and occasionally in gastric and colon adenocarcinomas [11]. To our knowledge, no available data exist on human hematologic neoplasias, other than Hodgkin lymphoma.

Given the compelling evidence of the importance of Tpl2/Cot in experimental and tissue culture models of T-cell neoplasias, we designed a study to investigate possible involvement of Tpl2/Cot in the pathogenesis of human T-cell neoplasias. Specifically, we studied 12 adults with various T-cell neoplasias to obtain a broad spectrum of T-cell malignancies, all with predominant leukemic expression, and examined whether Tpl2/Cot expression is deregulated in the transformed cells. The expression levels of Tpl2/Cot were quantitated by SybrGreen real-time RT-PCR using three different quantitation approaches (standard curve[12], absolute fluorescence increase [13] and the M.W.Pfaffl method [14]) as well as the conventional semi-quantitative RT-PCR.

## Results

### Evaluation of Tpl2/Cot mRNA expression in T-cell neoplasias

**Figure 1:**
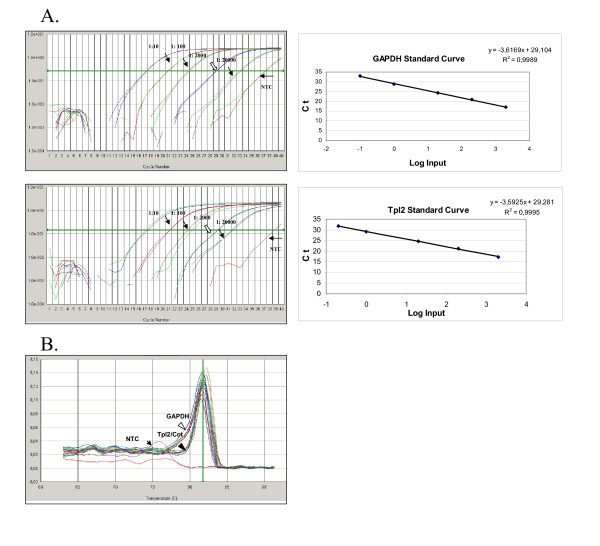
**Real-Time PCR validation A**. Serial dilutions of a standard cDNA duplicates for the construction of standard curves for GAPDH and Tpl2/Cot. The curve slopes shown on the upper right corner of each plot are -3.61 and -3.59 respectively. Black arrows correspond to dilution 1:10; white arrows to 1:20; left-pointed arrow is NTC (no-template control). X-axes represent the Log of the dilution factor, Y-axes the mean Ct of duplicates. **B**. Dissociation (melting) curve of the PCR products, showing a peak at 81.7°C for both Tpl2/Cot(black arrow) and GAPDH(white arrow), while NTCs have either no peak or a peak at a much lower temperature (thin arrow).

To determine the levels of expression of Tpl2/Cot mRNA we first established and validated a real time PCR approach. Melting curves showed that there were no by-products in both Tpl2/Cot and GAPDH reactions (Figure 1B). CVs of mean triplicate Ct (threshold cycle) ranged from 0.1% to 0.92% which account for a low intra-assay variability. A series of five 10 to 20-fold dilutions of a standard cDNA from different cDNA preparations were also run multiple times to determine primer efficiencies. Linear regression analysis of the standard curves [mean Ct plotted against the log(RNA input)] showed high linearity, with regression coefficients greater than 0.997 (Figure 1A). We used the standard curve slope in the equation

(1) E = 10^-1/slope^

to calculate the mean efficiency of Tpl2/Cot and GAPDH primers. The slopes were almost equal (from -3.59 to -3.62) for both primers which showed that we could use the Pfaffl method [14] without the need of a standard curve in every set of reactions. Specifically, to determine the ratio (R) of the normalized Tpl-2/Cot expression of sample vs control we used the equation



where Etarget and Eref are the Efficiencies of the target (Tpl2) and reference genes(GAPDH) respectively which both were equal to a mean of 1.89 and ÄCt is the difference between the mean Ct of control cDNA(CTR) and patient cDNA (Sample). To confirm our results we also tried the Absolute Fluorescence Increase method using the LinRegPCR software v.7.5 which measures the actual efficiency of each amplification curve by fitting its linear part in a simulation plot of the Log (fluorescence) versus Cycle and calculates the efficiency from the slope of a linear regression model of the simulation curve [13]. As control we used triplicates of cDNA from 3 healthy individuals, for which we calculated the mean Ct. The control samples were representative among 22 control specimens with similar values. Results were similar to those obtained by the Standard Curve method [12] (data not shown).

**Figure 2:**
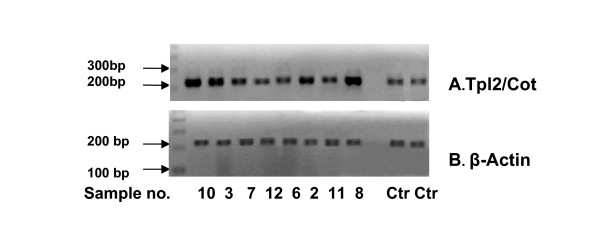
**Tpl2/Cot mRNA expression in T-cell neoplasms. **Representative semi-quantitative RT PCR for Tpl-2 mRNA expression in patients and controls. **A**. Tpl2/Cot PCR product of 228 bp **B**. β-Actin PCR product of 214 bp. Samples no 2, 3, 8 and 10 are overexpressed compared to the control and correspond to the LGL-PD patients shown in Table 1.

A total of 12 adult samples with T- and NK-cell neoplasias were analyzed according to the described method. They all had leukemic expression in the periphery. Morphology was assessed by light microscopy on peripheral blood smears, including measurement of absolute LGL number. Four out of twelve (33%) patients tested markedly overexpressed Tpl2/Cot (p = 0.034), as determined by either conventional RT-PCR or real-time qPCR (Tables 2 and 3, Figure 2). Interestingly, all the Tpl2/Cot overexpressing patients had LGL-PD, three with the phenotype of CD3+ T-LGL leukemia and one with the CD3- pattern of chronic NK-lymphocytosis. Three of these patients displayed neutropenia not attributable to BM infiltration, one in association with sarcoidosis (Table 1). The fourth patient with monoclonal T-LGL lymphocytosis had a co-current cutaneous T-cell lymphoma which, during follow-up, demanded systemic chemotherapy.

**Table 1:** Patient characteristics

**Patient no.**	**Disease**	**Sex**	**Age**	**%T+NKcells/PBMC**	**Disease state**	**Co-existent conditions**
1	SS	F	88	87	PP	
2	CTCL with monoclonal T- LGL lymphocytosis	M	63	86	RD	
3	T-LGL leykemia	F	75	93	RD	Neutropenia
4	MF	M	91	71	RD	
5	MF	M	85	71	RD	
6	TLL	F	35	77	RD	
7	T-PLL	M	85	67	RD	Myositis
8	Chronic NK -lymphocytosis	F	67	64	Stable for two years	Neutropenia
9	T-ALL	M	16	95 LB	RD	
10	T-LGL leukaemia with reactive NK lymphocytosis	F	60	71	Stable for 10 years	Sarcoidosis (past), neutropenia
11	PTCL secondary to MF	M	52	85	PR	
12	Pre-T-ALL	M	37	95 LB	RD	

Abbreviations: SS, Sezary syndrome; CTCL, Cutaneous T cell Lymphoma; T-LGL, T-large granular lymphocyte; MF, Mucosis Fungoides; NK, natural killer cell; TLL, T-lymphoblastic lymphoma; T-PLL, T-Prolymphocytic leukemia; T-ALL, T-acute lymphoblastic leukemia; LB, Lymphoblast ;PP, primary progressive; RD, recently diagnosed; PR, partial response.

**Table 2:** Tpl-2/Cot expression in PBMC

**Patient no.**	**Tpl2-Norm^a^**	**Fold increase (R)^b^**	**CV(%)-R^c^**
Ctr (n = 3)	0.04 ± 0.019	1.00 ± 0.119	45.3
1	0.02 ± 0.000	0.59 ± 0.009	1.6
2	0.13 ± 0.005	**3.88 **± 0.149	3.8
3	0.13 ± 0.005	**4.04 **± 0.143	3.5
4	0.02 ± 0.003	0.66 ± 0.092	13.9
5	0.04 ± 0.005	1.22 ± 0.092	11.8
6	0.06 ± 0.003	1.70 ± 0.085	5.0
7	0.06 ± 0.002	1.81 ± 0.076	4.2
8	0.17 ± 0.008	**5.21 **± 0.251	4.8
9	0.01 ± 0.000	0.32 ± 0.007	2.2
10	0.16 ± 0.006	**5.04 **± 0.194	3.8
11	0.04 ± 0.001	1.09 ± 0.037	3.4
12	0.04 ± 0.004	1.22 ± 0.108	8.9

a. Tpl2/Cot normalized to GAPDH by equation (3) in the text ± SD.

b. Tpl2/Cot-Norm (sample) ratio to Tpl2/Cot-Norm (control) by equation (2) in the text ± SD. Samples in which Tpl2/Cot is overexpressed are shown in bold.

c. % Coefficient variance of (R).

**Table 3:** Summary of the results

**Patient no.**	**Immunophenotype**	**Serum TNF-α (pg/ml)**	**Tpl2/Cot (fold increase)**
1	CD3+, CD4+, CD7-	1.7	0.6
2	CD3+, CD8+, CD56+, CD57+, TCRαβ+	1.9	**3.9**
3	CD3+, CD8+, CD16, 56-, CD57+, TCRαβ+	1.8	**4**
4	CD3+, CD4+, CD5+, CD7+	1.5	0.7
5	CD3+, CD4+, CD7-	1.9	1.2
6	TdT+, CD5+, CD2+	2.5	1.7
7	CD3+CD4+CD25-TCRγ+	3.8	1.8
8	CD2+, CD3-, CD16+, CD56-, CD57-, TCR-	6.2	**5.2**
9	TdT+, CD2+, CD3+, CD7+, CD4-, CD8-	2.6	0.3
10	2 populations: a) T-LGL CD3+, CD8+, CD56+, CD57+, TCR γ+ b) NK CD2+, CD3-, CD16, 56+, 57-	1.9	**5**
11	CD3+, CD4+, CD7-, CD25-, TCRαβ+	1.2	1.1
12	TdT+, cCD3+, CD7+, CD4-CD8-	1.5	1.2

### Evaluation of serum TNF-α and IL-2 levels

Overexpression of Tpl2/Cot in Jurkat T-cells induces TNF-α expression [7]. Tpl2 also regulates TNF-α expression in macrophages by activating ERK and thus controlling the posttranscriptional modification of the TNF-α mRNA, which is necessary for its export from the nucleus [15]. We, therefore, evaluated serum TNF-α levels in all patients studied. In order to have an internal negative control in the study we analyzed 8 samples from normal blood donors. The mean TNF-α concentration in patient sera was 2.37 ± 1.4 pg/ml with a range between 1.2 pg/ml and 6.2 pg/ml, while in the control group it was 0.6 ± 0.2 pg/m. The mean patient TNF-α value was statistically significant higher than the respective of the healthy controls (p = 0.002), as was TNF-α in the LGL-PD group alone compared to the control group (p = 0.006). It is of interest that the patient with chronic NK-lymphocytosis and neutropenia displayed the highest TNF-α value (6.2 pg/ml) that was associated with the highest Tpl2/Cot expression (5.2 fold compared to control) (Table 3) suggesting a relationship between Tpl2/Cot overexpression and TNF-α overproduction in this patient. There was no difference in TNF-α levels between the group of LGL-PD patients and the group of patients with the remaining T-cell other neoplasias (p = 0,392).

Tpl2/Cot overexpression in Jurkat and EL-4 T-cells induces IL-2 secretion in culture. We, therefore, examined the expression levels of IL-2 in the sera from the 12 patients studied and 8 control samples. No circulating IL-2 was detected in the sera of the patients or the control donors (data not shown).

### Overexpression of Tpl2/Cot in LGL-PD is not associated with gene amplification

**Figure 3:**
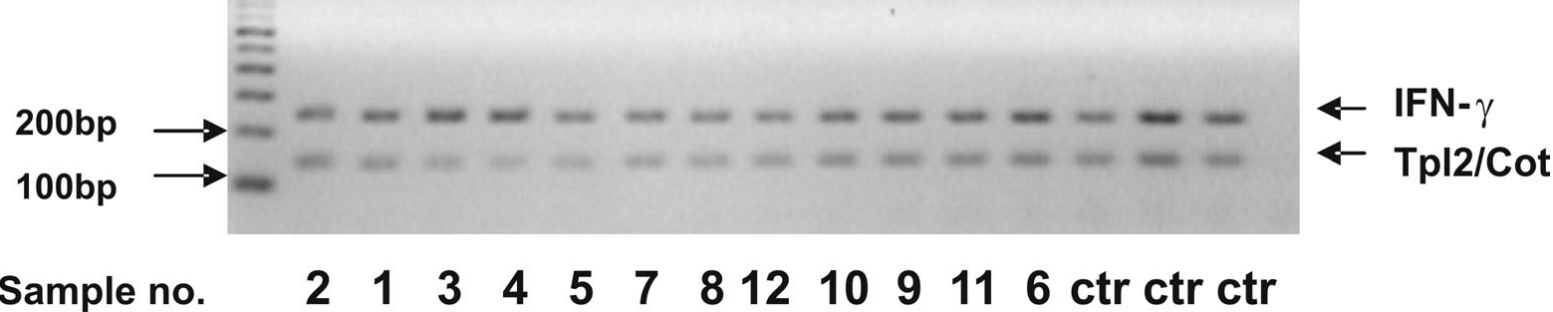
**The *tpl2*/*cot *genomic locus is not amplified in the T-cell neoplasms analyzed. **Multiplex PCR for the quantification of the *Tpl-2*/*Cot *gene load, relative to *IFN*-γ gene in patient and control DNA: *tpl2*/*cot *PCR product detected at 139 bp; *IFN*-γ PCR product at 250 bp. No significant difference between samples is evident.

Overexpression of Tpl2/Cot in human breast cancer has been associated with amplification of the *tpl2 *genomic locus [9]. We, thus, evaluated whether the overexpression of Tpl2/Cot in T-cell malignancies is associated with amplification of the genomic *tpl2 *locus. For this purpose genomic DNA was isolated from PBMCs of the same patients and part of the genomic *tpl2 *locus was amplified using multiplex PCR. As reference gene we used the *IFN*-γ gene. The results showed a similar *tpl2*/*IFN*-γ ratio in all cases (Figure 3) indicating that overexpression of Tpl2/Cot in LGL-PDs is not due to gene amplification.

## Discussion

Experimental data based on in vitro and animal model have shown that Tpl2/Cot is an important regulator in the transduction of signals leading to T-cell activation [16]. Overexpression of this kinase results in increased proliferation of T-cells by activating E2F-dependent transcriptional activity [8]. A truncated form present in rodents exhibits increased catalytic activity, and when overexprerssed as a T-cell-specific transgene in mice it induces tumors within 3–9 months [3]. Tpl2/Cot activates the transcription factors NFAT and NFkB in T-cells, which drive the transcription of several cytokine genes such as IL-2 and TNF-α [4,7]. In macrophages, Tpl2/Cot is essential for the activation of ERK by LPS via TLR4 and the export of TNF-α mRNA from the nucleus [15]. The preceding evidence supports a possible involvement of Tpl2/Cot in human T-cell neoplasias.

Investigation of Tpl2/Cot expression in human tumor specimen has shown that it is occasionally overexpressed in colon and gastric adenocarcinomas [11] and human breast cancer tissues [9]. Tpl2/Cot overexpression has also been detected in a hepatocellular carcinoma cell line [17] and in patient tumor tissue from EBV-related Hodgkin lymphomas and nasopharyngeal carcinomas[10]. In the present study we analyzed peripheral blood from patients with T- and NK-cell lymphoproliferative diseases at the time where the patients were not receiving any treatment and had profound leukemic expression in the periphery. Out of the 12 cases analyzed, Tpl2/Cot was found overexpressed in three T-LGL-leukemias and in one chronic NK-lymphocytosis accounting for all four LGL-PDs studied. These findings suggest that Tpl2/Cot deregulation may be a defining molecular event for the development of this type of neoplasias. Given the role of Tpl2/Cot in T-cell proliferation via activation of E2F transcription [8], overexpression of this kinase may contribute to neoplastic cell proliferation.

Large granular lymphocyte lymphoproliferative diseases (LGL-PD) are relatively rare and not well defined disorders, frequently associated with autoimmune diseases or immune mediated manifestations such as rheumatoid arthritis (pseudo-Felty syndrome [18]), RF positivity, neutropenia and pure red cell aplasia [19,20]. They present clinical, morphological and immunological distinct features, resulting from chronic proliferation of CD3+ or CD3- granular lymphocytes. In the CD3+ cases the proliferating cells express CD8 and NK-associated surface antigens such as CD16, the LGL-specific CD57 antigen and CD45RA, and display also clonal rearrangement of the TCR alpha-beta or, less often, gamma-delta chains, thus representing cytotoxic effector T-cells[21]. The T-LGL leukemias are by definition indolent, but there are rare reports indicating evolution to high grade lymphoma [22]. Limited data on recurrent chromosomal aberrations exist [23,24]. In the rare CD3- cases the cells are TCR-, CD2+, CD16+ and CD56+ representing, therefore, true NK-cell proliferations corresponding to the aggressive NK-cell leukemias or to the – usually benign – chronic NK-lymphocytosis [19,20]. Lack of Tpl2/Cot gene amplification in our LGL-PD patients indicate that overexpression is either due to changes in the regulation of Tpl2/Cot gene activation or mRNA stability. Such changes can be either primary (i.e. mutations that affect the promoter or the mRNA stability) or secondary (i.e. activation of transcription factors that affect the Tpl2/Cot promoter or signaling molecules that affect the stability of its mRNA).

There is accumulating evidence suggesting that patients with LGL-PDs display frequently immune manifestations and increased TNFα production by the neoplastic T-cells has been reported to play a role in their pathogenesis[20,25]. Interestingly, three of our LGL-PD patients displayed neutropenia not attributable to BM infiltration while one of these patients displayed also sarcoidosis. Evaluation of circulating TNF-α level in the patients showed that the highest TNF-α value was found in the LGL-PD patients that displayed also the highest Tpl2/Cot expression among the subjects studied. These findings are in agreement with previous reports demonstrating that Tpl2/Cot is involved in TNF-α expression and secretion [7,15] while provide evidence for a causal relationship between the Tpl2/Cot overexpression, the TNF-α overproduction and the pathogenesis of neutropenia in LGL-PD patients [25]. TNF-α was increased in the sera of patients where Tpl2/Cot expression was not elevated, indicating that in these patients TNF-α may be upregulated via alternative pathways not associated with overexpression of Tpl2/Cot.

## Conclusions

In conclusion, data from the present study suggest that Tpl2/Cot overexpression may have a role in the development of certain types of human T-cell neoplasms thus confirming experimental data on animal and tissue culture models for the role of Tpl2/Cot in T-cell malignancies.

## Methods

### Patients

Peripheral blood samples from 12 adults aged 16–88 years (median age 64 years) with various T- and NK-cell neoplasias with peripheral blood leukemic expression were collected during a two-year time period. Patients with signs of infection or recently subjected to cytotoxic therapy were excluded from the study. Diagnosis was based on morphological, immunophenotypic and genomic studies and histological findings of bone marrow and/or lymph node biopsies and disease was classified according to the WHO classification [26]. There were three patients with T-LGL leukemia, one patient with chronic NK lymphocytosis, one patient with Sezary syndrome (SS), two patients with Mucosis Fungoides (MF), one patient with T-Prolymphocytic Leukemia (T-PLL), two patients with T-acute Lymphoblastic Leukemia (T-ALL), one patient with T-lymphoblastic Lymphoma (TLL) and one patient with Peripheral T-Cell Lymphoma (PTCL) secondary to MF. Detailed patient characteristics are presented in Table 1. Complete blood counts and flow cytometric analysis of peripheral blood lymphocytes were performed at the day of blood collection for the molecular study. In addition, patient sera were obtained by centrifugation of 4 ml of non-anticoagulated blood at 3000 rpm for 10 min and were stored at -80°C for IL-2 and TNF-α measurement. Peripheral blood specimens from 22 healthy volunteers age- and sex-matched with the patients were collected and used as controls. This research project was subjected to and approved by the Ethics Committee of the University Hospital of Heraclion.

### Peripheral Blood Mononuclear Cell isolation and RNA extraction

Peripheral blood mononuclear cells (PBMC) were isolated from 9 ml of fresh EDTA-K_3 _anti-coagulated peripheral blood samples by Lymphoprep density centrifugation (Nycomed Pharma AS, Norway). PBMCs were immediately lyzed in suitable volume of Trizol LS reagent (Invitrogen, UK) and mRNA was isolated according to the manufacturers' protocol. An aliquot of 1 μg of total RNA was treated with 1 IU DNase I, Amplification Grade (Invitrogen, UK) to eliminate any traces of genomic DNA.

### Semi-quantitative RT-PCR

First strand cDNA was synthesized by reverse transcription of 1 μg total RNA using the Thermoscript™ RT kit (Invitrogen, UK). 0.8 μl of cDNA were amplified in a 20 μL PCR reaction containing 250 nM of each primer, 200 nM dNTPs, 0.5 IU Taq polymerase and 2 μl of 10X reaction buffer (Platinum Taq DNA Polymerase kit, Invitrogen, UK). Reactions were first optimized for annealing temperature, Mg and primer concentration (data not shown). Primers for Tpl2/Cot detection were derived from exons 3 and 4 of the human Tpl2/Cot gene (Genbank accession no: AL547407), spanning an 8.5 kb intron to prevent co-current genomic DNA amplification and their sequences were: forward 5'-CAG TAA TCA AAA CGA TGA GCG TTC TAA-3', reverse 5'-GAA CAT CGG AAT CTA TTT GGT AAC GTC-3' producing a 228 bp-length amplicon. For normalization of mRNA input differences human beta actin mRNA (Genbank accession no: BC013835) was detected using the following primers: forward 5'-CCG GCC AGC CAG GTC CAG A-3', reverse 5'-CAA GGC CAA CCG CGA GAA GAT G-3', amplifying a 214 bp cDNA fragment. In each reaction two negative controls were included by either omitting reverse transcriptase at the RT step or cDNA template respectively.

PCR reactions were performed on a thermal cycler (PTC-200 MJ Reasearch with heated lid) and repeated 3 times with different cDNAs from the same mRNA. Expression of Tpl2/Cot was determined by semi-quantitative, relative RT-PCR. An amplification curve for each gene was acquired by performing the reaction with increasing number of PCR annealing cycles to identify the exponential phase of the reaction. The thermocycling parameters were as follows: initial denaturation at 94°C for 5 min followed by 34 for Tpl2 or 23 for actin cycles at 94°C for 30 sec, 54°C for 30 sec, 72°C for 30 sec and a final extension at 72°C for 7 min.

The RT-PCR products were analysed by electrophoresis in a 2.5% agarose gel, stained with 0.2 μg/ml ethidium bromide and visualized in a UVP transiluminator (Gel Doc 1000 Bio Rad). The band intensity was analysed by a densitometric image analysis system (TINA scan v2.07) and the results were expressed as a ratio between Tpl2/Cot and β-actin band intensity.

### Real-time PCR

Primers for real-time PCR were designed with the Primer Express Software v.2 (Applied Biosystems) and selected so that they amplify a region of no more than 150 bp, they have similar GC content, same Tm, no more than 3 G or C's at the 3' end and no secondary structure formation. To exclude primers with more than 3 consecutive complementary bases between them we used Qiagen Oligo Analysis & Plotting Tool (Qiagen, Germany). Primers forTpl2/Cot annealed to exons 6–7 and their sequences were: forward 5'-TCC TAA GGA CCT CCG AGG AAC-3', reverse 5'-CCC AGG CTG TAG ATG TCT GCT-3', amplifying a 93 bp region. GAPDH was used as a reference gene to compensate for mRNA input differences. Primers for GAPDH derived from exons 2–3 (Genbank accession no: BC023632) and their sequences were: forward 5'-GGA AGG TGA AGG TCG GAG TCA-3', reverse 5'-GTC ATT GAT GGC AAC AAT ATC CAC T-3', amplifying a 101 bp region.

Primer concentration, cDNA dilution, Mg concentration and annealing temperature were optimized so that a maximum fluorescent signal with no inhibition from RT components and a similar reaction yield from both primer sets could be obtained. For the real-time PCR study cDNA from first strand synthesis treated with RNase H for 20' at 37°C was diluted 1:20 with DNAse-free water and 5 μl were used in a 20-μl reaction mixture consisting of 10.4 μl 2x SybrGreen PCR Master mix, 6 mM final concentration of MgCl_2 _and 500 nM of the Tpl2/Cot primers or 100 nM of GAPDH primers. Reactions were carried out using an ABI Prism 7000 sequence detector (Applied Biosystems, Foster City, CA, USA) according to manufacturer's instructions. The thermal profile used consisted of 2 min at 50°C,10 min at 95°C and 40 repeats of denaturation at 95°C for 15 sec and annealing-extension-fluorescence data acquisition at 60°C for 1 min. A post-PCR Melting Curve Analysis was performed by a 20-min slow ramp from 60° to 95°C to confirm that there were no by-products. Samples were run in a 3% agarose gel after the end of reaction to confirm specificity. All samples were run in triplicates and two negative controls with either no reverse transcriptase or no cDNA template were included. Reaction was repeated twice in different days to estimate inter-assay variation. Results were analyzed using the ABI Prism 7000 SDS software (v.1.1, Applied Biosystems) and Excel for further quantitative study.

### DNA extraction and multiplex PCR

To detect possible gene amplification in cases with Tpl2/Cot overexpression we used a multiplex DNA PCR protocol with interferon-γ (IFN-γ) as a reference gene amplified at the same reaction tube. High molecular weight DNA was isolated from PBMC by proteinase K digestion and phenol chloroform extraction with ethanol precipitation and diluted in TE buffer. The primers for Tpl2/Cot were: forward 5'-GCG ACG GAT TGA GGT TTG-3', reverse 5'-GCG TTT CAG GCG TAT GGA-3' amplifying a 139 bp region of intron 1(Genbank accession no.AY309013) and the primers for IFN-γ were: forward 5'-ATG CAG GTC ATT CAG ATG TAG C-3', reverse 5'-TTG GAT GCT CTG GTC ATC TTT A-3' amplifying a 250 bp fragment containing intron and exon sequences between exons 2–3 (Genbank accession no: J00219). 50 ng of DNA were used in a 20 μl reaction containing 10 μl Platinum qPCR Supermix-UDG (Invitrogen, UK), 250 nM Tpl2/Cot primers and 100 nM IFNγ primers. The number of amplification cycles was adjusted so that the reaction terminated at the middle of the exponential phase of both products (data not shown). The thermal profile consisted of a denaturation step at 95° for 10 min, followed by 29 repeats at 95°C for 15 sec, 58°C for 30 sec and 72°C for 30 sec and a final extension at 72°C for 7 min. PCR products were analysed in a 3% agarose gel, visualized and scanned as described earlier and the Cot/IFNγ ratio was determined.

### Peripheral blood lymphocyte immunophenotype and assessment of T-cell clonality

Two-color flow cytometry was used for the analysis of peripheral blood lymphocyte subpopulations. In brief, 100 μL aliquots of EDTA-anticoagulated peripheral blood samples were surface stained with each of the following PE- or FITC-conjugated mouse antihuman monoclonal antibodies: anti-CD2, anti-CD3, anti-CD4, anti-CD8, anti-CD5, anti-CD7, anti-CD16, anti-CD56, anti-CD57, anti-CD19, anti-CD25, anti-CD11b, anti-CD79a, anti-FMC7 and anti-HLA-DR (Beckman Coulter, France). Cells were also stained for intracellular Terminal deoxy-transferase (TdT) (Beckman Coulter) and CD3 using the IntraPrep intracellular staining kit (Beckman Coulter). PE- or FITC-conjugated mouse IgG of appropriate isotype served as negative controls. Following 30 min incubation at room temperature and two washes with phosphate buffer saline (PBS)-1% fetal bovine serum (FBS)-0.05% azide, contaminating red cells were lysed with 0.12% formic acid and samples were fixed in 0.2% parafolmadeyde using the Q-prep reagent system (Coulter, Louton, England). Analysis on 10,000 events was performed in an Epics Elite model flow cytometer (Coulter) in the lymphocyte gate.

Clonality assessment of peripheral blood T-cells was performed by analysing quantitatively different variable regions of the T-cell receptor (TCR) β chain (Vβ repertoire) of CD3^+ ^cells by means of flow cytometry using the IOTest Beta Mark kit (Beckman Coulter), according to the manufacturer's instructions [27]. T-cell clonality assessment was also performed by PCR analysis using primers for the TCR V(D)J junction in PBMC derived DNA, according to standard methods in a reference laboratory.

### ELISA

TNF-α concentration was determined using the High Sensitivity human TNF-α ELISA kit (R&D, USA) or the IL-2 ELISA kit (R&D, USA) according to the manufacturer's instruction. According to the manufacturer, the sensitivity of these assays are 0.12 pg/ml and 7 pg/ml respectively.

### Statistical analysis

The comparison of Tpl2/Cot mRNA expression between patient and control samples was performed by means of the nonparametric Mann-Whitney U test using as variables the mean normalized Tpl-2 expression of each sample and control, described by the equation

(3) Tpl-2 norm = (E)^-(mean CtTpl2-meanCtGAPDH)^

where E is the mean common efficiency of the reference and target gene that we calculated (E = 1.89, see section Results)[14]. The Mann-Whitney U test was also used for the comparison of the TNF a and IL-2 levels between the Tpl-2 overexpressing patients, the controls and the non-overexpressing patients.

## Authors' contributions

AVC collected specimens and patient data and performed the major body of the experimental work as well as the data analysis and the preparation of the manuscript. HAP was involved in flow cytometric analysis, patient selection and manuscript preparation. ANM and GDE were involved in the study design, the interpretation and evaluation of the clinical data and manuscript preparation. CT conceived the project, performed the ELISAs, was responsible for the coordination of the experimental procedures and was involved in the preparation of the manuscript.
